# The Dunn Worry Questionnaire and the Paranoia Worries Questionnaire: new assessments of worry

**DOI:** 10.1017/S0033291719000588

**Published:** 2020-04

**Authors:** Daniel Freeman, Jessica C. Bird, Bao S. Loe, David Kingdon, Helen Startup, David M. Clark, Anke Ehlers, Emma Černis, Gail Wingham, Nicole Evans, Rachel Lister, Katherine Pugh, Jacinta Cordwell, Graham Dunn

**Affiliations:** 1 Department of Psychiatry, University of Oxford, Oxford, UK; 2 Oxford Health NHS Foundation Trust, Oxford, UK; 3 The Psychometrics Centre, University of Cambridge, Cambridge, UK; 4 Academic Department of Psychiatry, Faculty of Medicine, University of Southampton, Southampton, UK; 5 Sussex Partnership NHS Trust, Worthing, UK; 6 Department of Experimental Psychology, University of Oxford, Oxford, UK; 7 Oxford University Hospitals NHS Foundation Trust, Oxford, UK; 8 Centre for Biostatistics, Institute of Population Health, University of Manchester, Manchester, UK

**Keywords:** Item response theory, paranoia, questionnaire development, worry

## Abstract

**Background:**

The cognitive process of worry, which keeps negative thoughts in mind and elaborates the content, contributes to the occurrence of many mental health disorders. Our principal aim was to develop a straightforward measure of general problematic worry suitable for research and clinical treatment. Our secondary aim was to develop a measure of problematic worry specifically concerning paranoid fears.

**Methods:**

An item pool concerning worry in the past month was evaluated in 250 non-clinical individuals and 50 patients with psychosis in a worry treatment trial. Exploratory factor analysis and item response theory (IRT) informed the selection of scale items. IRT analyses were repeated with the scales administered to 273 non-clinical individuals, 79 patients with psychosis and 93 patients with social anxiety disorder. Other clinical measures were administered to assess concurrent validity. Test-retest reliability was assessed with 75 participants. Sensitivity to change was assessed with 43 patients with psychosis.

**Results:**

A 10-item general worry scale (Dunn Worry Questionnaire; DWQ) and a five-item paranoia worry scale (Paranoia Worries Questionnaire; PWQ) were developed. All items were highly discriminative (DWQ *a* = 1.98–5.03; PWQ *a* = 4.10–10.7), indicating small increases in latent worry lead to a high probability of item endorsement. The DWQ was highly informative across a wide range of the worry distribution, whilst the PWQ had greatest precision at clinical levels of paranoia worry. The scales demonstrated excellent internal reliability, test-retest reliability, concurrent validity and sensitivity to change.

**Conclusions:**

The new measures of general problematic worry and worry about paranoid fears have excellent psychometric properties.

## Introduction

Excessive worry is identified as a contributory causal factor in many mental health disorders, including anxiety (Borkovec and Inz, 1990), depression (Watkins, 2008), eating disorders (Sternheim *et al*., 2012) and persecutory delusions (Freeman, 2016). Our view is that worry brings fearful ideas to mind, keeps them there and elaborates the content. Distress escalates and feared outcomes are judged as more likely to occur. We consider problematic worry to comprise: repeated thinking about problems that cause anxiety about the future; a focus on potential things that could go wrong; problems being catastrophised; a belief of lack of control over the thinking process; and interference in activities and distress. We wished to develop a new measure that assesses current levels of problematic worry across the spectrum of severity in the population. Importantly, we wanted a high level of clarity in item content and ease of use, basing the format on the successful Warwick Edinburgh Mental Well-being Scale (Tennant *et al*., 2007).

The most commonly used measure of worry, the Penn State Worry Questionnaire (PSWQ; Meyer *et al*., 1990), was developed 30 years ago. The questionnaire was derived from a principal component analysis of an item pool completed by 300 psychology students. It helped initiate the psychological study of worry and has been used with thousands of people in research studies and clinical trials, showing good test-retest reliability, internal reliability and convergent and discriminant validity. It is a trait measure of worry, comprising 16 items rated on a 1–5 scale with anchors at each end (‘Not at all typical of me’/‘Very typical of me’). Five items are reverse worded, a procedure that can be problematic due to participant inattention and confusion (Woods, 2006; Van Sonderen *et al*., 2013). The reverse items on the PSWQ tend to produce a separate artefact factor (Brown, 2003; Yilmaz *et al*., 2008). Our own experience is that the reverse items on the PSWQ can sometimes confuse people. The PSWQ items focus upon a tendency to worry (e.g. ‘I have been a worrier all my life’, ‘I am always worrying about something’, ‘I never worry about anything’). There are no items on the emotional impact of worry. Not every scale item is easily comprehendible (e.g. ‘If I do not have enough time to do everything, I do not worry about it’). The overall success of the scale has led to adaptations, including shorter versions and state versions (e.g. Yao *et al*., 2016).

Our objective was to produce – using a latent trait model approach with item response theory (IRT) (Reise and Henson, 2003) – a new worry scale, straightforward to complete, that focuses on problematic worry. IRT examines the probabilistic relationship between varying levels of a latent trait and the ability of individual items to measure this trait. By aligning items and respondents on the same scale, IRT leads to the development of measurements with greater precision and parameters that are sample independent (Bortolotti *et al*., 2013). Our aim was to develop a general worry scale that would: assess worry over a defined time period (1 month); be brief; use a scale with anchors for every point; not include reverse items; include the tendency to worry but also levels of control and emotional impact; and would be suitable for use in clinical and non-clinical populations. The scale was designed to be neutral with regards to theoretical accounts of the causes of worry (Davey and Meeten, 2016). Our secondary objective was to develop a content-specific measure of worry focused upon paranoid concerns (unfounded fear of harm from others). Previously we have shown that: patients with persecutory delusions have levels of worry comparable to individuals with generalised anxiety disorder (GAD) (Freeman and Garety, 1999); worry predicts the occurrence and persistence of paranoia (Freeman *et al*., 2012); and treating worry in patients with persecutory delusions significantly lowers the delusions [The Worry Intervention Trial (WIT); Freeman *et al*., 2015]. In the WIT, we independently assessed levels of general worry and paranoia. For the implementation of such worry treatments for patients with psychosis, it will be beneficial for clinicians to have a brief measure that combines both concepts. We also planned to produce clinical cut-off scores for the questionnaires to facilitate use as screening tools.

## Method

### Participants

To extract the items for the new measures of general worry and worry concerning paranoia, a derivation sample of 300 participants (250 from the general population and 50 patients with persecutory delusions) completed the full item pool (mean age = 42.8, s.d. = 18.6, female = 167, male = 133, White British = 90%). A second cross-validation sample consisting of 449 participants [273 from the general population, 79 patients with persecutory delusions and 93 patients with social anxiety disorder (SAD)] completed the final versions of both measures (mean age = 33.5, s.d. = 13.8, female = 192, male = 257, White British = 90%).

Participants from the general population were recruited via local radio adverts and the distribution of leaflets in Oxfordshire. Patients with persecutory delusions were participants from the WIT, a randomised controlled trial of a psychological intervention to reduce worry in adults with persecutory delusions in the context of non-affective psychosis (Freeman *et al*., 2015). They had: a clinical diagnosis of non-affective psychosis (i.e. schizophrenia, schizoaffective disorder or delusional disorder); a current and persistent persecutory delusion; and clinically significant levels of worry (44+ on the PSWQ; Startup and Erickson, 2006). Participants with SAD were referred for psychological treatment by their GP or IAPT services to either the London or Oxford Centre for Anxiety Disorders and Trauma. They met criteria for SAD according to the Anxiety and Related Disorders Interview Schedule for DSM-IV (Brown *et al*., 1996) and SAD was their primary clinical problem.

### Assessments

#### Item pools

An initial item pool of 40 general worry items was devised by the study team based upon consideration of our definition of worry, comments patients had made, clinical experience and existing questionnaires. We aimed to cover items concerning time spent worrying, control over worry and the impact of worry. An initial item pool of 16 paranoia worry items was created from patient comments during the WIT and the team's clinical experience. The time period for all the items was 1 month. Following the response format of the WEMSBS (Tennant *et al*., 2007), items were rated on a 0–4 scale (None of the time, Rarely, Some of the time, Often, All of the time).

#### Penn State Worry Questionnaire

The PSWQ is the most established measure of trait worry and has been used in non-clinical and clinical populations (Meyer *et al*., 1990; Startup and Erickson, 2006). Each of the 16 items is rated on a five-point scale. Higher scores indicate a greater tendency to worry.

#### Perseverative Thinking Questionnaire

This is a 15-item questionnaire asking how a person typically thinks about negative problems (e.g. ‘The same thoughts keep going through my mind again and again’), with each item assessed on a 0–4 scale (Ehring *et al*., 2011). Higher scores indicate greater levels of repetitive negative thinking.

#### Green *et al*. Paranoid Thoughts Scale Part B

The Green *et al*. Paranoid Thoughts Scale (GPTS) Part B is a 16-item measure of persecutory thinking (e.g. ‘I was convinced there was a conspiracy against me’) (Green *et al*., 2008). Items are rated on a 1–5 scale. Higher scores indicate greater levels of paranoid thinking.

#### Beck Anxiety Inventory

The Beck Anxiety Inventory (BAI) is a self-report 21-item assessment of anxiety. Items are rated on a 0–3 scale (Beck *et al*., 1988). Higher scores indicate higher levels of anxiety.

#### Depression Anxiety Stress Scales

The Depression Anxiety Stress Scales (DASS Anxiety) subscale comprises 14 items rated on a 0–3 scale (Lovibond and Lovibond, 1995). Higher scores indicate higher levels of anxiety.

#### Generalised Anxiety Disorder Questionnaire-IV

The Generalised Anxiety Disorder Questionnaire-IV (GAD-Q-IV) is a nine-item self-report diagnostic measure for GAD (Newman *et al*., 2002). A cut-off score of 7.67 is recommended to indicate probable GAD diagnostic status (Moore *et al*., 2014). This was used to from a GAD subgroup (*n* = 50) from the non-clinical participants.

#### Positive and Negative Syndrome Scale

The Positive and Negative Syndrome Scale (PANSS) is a 30-item interviewer-rated instrument developed for the assessment of patients with schizophrenia (Kay, 1991). Current symptoms over the last week were rated. Higher scores indicate the greater presence of psychiatric symptoms. Only the general psychopathology scale was considered in this study, as a marker of changes in levels of affect.

### Procedure

Participants from the general population completed questionnaires online using Qualtrics and participants from the clinical samples completed paper versions of the questionnaires. The derivation sample (*n* = 300) completed the full item pools for both measures. The final versions of both measures were then completed by the cross-validation sample (*n* = 449). To assess concurrent validity in the cross-validation sample, participants from the general population also completed the PSWQ, GAD-Q-IV, Perseverative Thinking Questionnaire (PTQ), DASS and the GPTS, and the persecutory delusion group completed the PSWQ, BAI, GPTS, PTQ and PANSS. No additional measures were completed by the patients with SAD. To examine test-retest reliability, 75 participants from the cross-validation sample (50 from the general population and 25 with persecutory delusions) also repeated the new measures 1 week later. To assess sensitivity to clinical change, the worry scales were repeated by 43 participants (who had been in the cross-validation sample) with a persecutory delusion during the WIT (Freeman *et al*., 2015).

### Analysis

All analyses were conducted in R, version 3.5 (R Core Team, 2013). Rates of missing data were low (<3%). Participants with missing data on the item pools were excluded (derivation sample *n* = 8, cross-validation sample *n* = 4). Demonstrating the appropriateness of factor analysis in the derivation sample, Bartlett's test of Sphericity was significant (χ^2^ = 22 577, df = 1540, *p* < 0.001) and the Kaiser–Meyer–Olkin test of sampling adequacy was excellent (KMO = 0.98). To assess the factor structure and separability of the items, an exploratory factor analysis (EFA) with the maximum likelihood estimator and oblique rotation was conducted using the ‘Psych’ package (Revelle, 2018). Parallel analysis and examination of the scree plot was used to identify the number of factors to extract from the 56 items. The EFA and subsequent IRT analyses were used to inform the selection of items for the final versions of both scales.

The sample sizes were sufficient for IRT analysis given previous recommendations that a minimum of 250 will provide stable estimates of the item parameters for questionnaire development (Orlando-Edelen and Reeve, 2007). Given the polytomous response options, for each measure a two-parameter graded response model (Samejima, 1969) IRT analysis was conducted using the ‘mirt’ package (Chalmers, 2012). Items with poor item fit (signed χ^2^ test of *p* < 0.01; Orlando and Thissen, 2000) or residual correlations above +0.2 (Yen, 1993) were excluded.

The IRT analysis produced discrimination and difficulty parameters for each item. The *θ* values represent the number of standard deviations from the average level (*θ* = 0) of the latent trait (i.e. general worry or paranoia worry), with higher values representing more severe presentations. Discrimination (*a*) parameters represent the capacity of each item to discriminate among participants at different levels of severity (i.e. *θ*). Higher discrimination values indicate the probability of endorsing the item increases rapidly as the level of severity increases. Discrimination values of at least 0.5 are considered acceptable, whilst values above 1 are highly discriminative (Baker and Kim, 2017). For each item, the difficulty parameters (*b*) represent the 50% probability of responding at the threshold between each option (*b*_1_ = 0–1, *b*_2_ = 1–2, *b*_3_ = 2–3 and *b*_4_ = 3–4). Higher levels of difficulty represent items that measure the severe end of the spectrum.

To validate the psychometric properties of the selected items for both questionnaires, the IRT analyses were repeated in the cross-validation sample. The overall test information (TI) provides a measure of the internal reliability of the scale across the *θ* distribution. For interpretability, TI at specific values of *θ* was converted into an equivalent *α* reliability using the formula 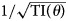 (O'Connor, 2017). The concurrent validity of the two scales was examined by evaluating the pattern of correlations between each scale and additional measures relating to worry, anxiety and paranoia and differences in the total scores between the participant subgroups. For the group comparisons, participants from the general population were split into those scoring above (*n* = 50) and below the clinical threshold of 7.67 on the GAD-Q-IV.

To assess sensitivity to change in the 43 patients with psychosis from the cross-validation sample, the mean change in scores between the two time points was examined and effect sizes calculated using the formula (*M*_pre_-*M*_post_)/*SD*_pre_. Individual changes on the scales were assessed using the reliable change index (RCI; Jacobson and Trux, 1991) in the 12 participants from this sub-group who completed the worry questionnaires directly before and after receiving a worry treatment (Freeman *et al*., 2015). For the RCI, Cronbach's *α* was calculated for each measure from the cross-validation sample of patients with persecutory delusions (*n* = 79).

To examine the ability of each measure to accurately identify clinical levels of general worry and paranoia worry receiver operating characteristic (ROC) analyses were conducted using the ‘pROC’ (Robin *et al*., 2011) and ‘optimumCutpoints’ (Lopez-Raton *et al*., 2014) packages. Patients in the WIT were an appropriate discrimination group given they had been identified based on reliably rated clinical levels of worry and desire for an intervention to reduce worry. To remove potential cases of clinical worry from the general population in the general worry analysis, participants from the non-clinical population who scored above cut-off on the GAD-Q-IV formed a GAD subgroup that were excluded from this analysis (*n* = 51). ROC curves were generated with the area under the curve (AUC) indicating the measure's discriminatory power, with values above 0.70 considered fair, over 0.80 good and over 0.90 excellent (Egan, 1975). The optimal clinical cut-off threshold, calculated based on Youden's *J* statistic (Youden, 1950), represents the optimal balance of sensitivity and specificity for the accurate discrimination of cases whilst reducing rates of false positives/negatives. The R code for the IRT and ROC analysis is available in the online Supplementary materials.

## Results

### Extracting the questionnaires

An initial EFA of all 56 items in the derivation sample identified two distinct factors for general worry and paranoia worry. To obtain a clean factor structure, one paranoia worry item that primarily loaded on the general worry factor and two items (one item from each factor) with cross-loadings over 0.3 were deleted. EFA of the remaining 53 items supported a two-factor structure which explained 73% of the variance. Although the two factors were highly correlated (*r* = 0.73), the distinct factor structure demonstrated that general worry (39 items) and paranoia worry (14 items) were separable. The two scales were therefore treated as unidimensional measures and IRT analyses were conducted on the general worry and paranoia worry items separately. The factor loadings and item parameters for the initial IRT analyses are shown in online Supplementary materials Table S1.

### General worry items

Following the initial removal of items with correlated residuals (18 items), IRT analysis was conducted. All 21 remaining items were highly discriminative with parameters ranging from 1.62 to 4.88 (online Supplementary materials). Preference was given to items with a range of difficulty thresholds to ensure the questionnaire would represent a wide proportion of the distribution of worry. Ten final items were selected to represent the theoretically important aspects of worry: time spent worrying (two items), control over worry (two items), interference of worry (two items) and the emotional consequences of worry (four items).

### Paranoia worry items

Following removal of items with correlated residuals (five items), IRT analysis was conducted. All nine remaining items were extremely discriminative with parameters ranging from 4.33 to 10.0 (online Supplementary material 1). All items demonstrated high difficulty parameters indicating that they tended to discriminate at more severe levels of paranoia worry. Five items were selected based on time spent worrying (two items), control over worry (one item), interference of worry (one item) and the emotional consequences of worry (one item). The final scales can be seen in the Appendix.

### Cross-validation

**Fig. 1.**
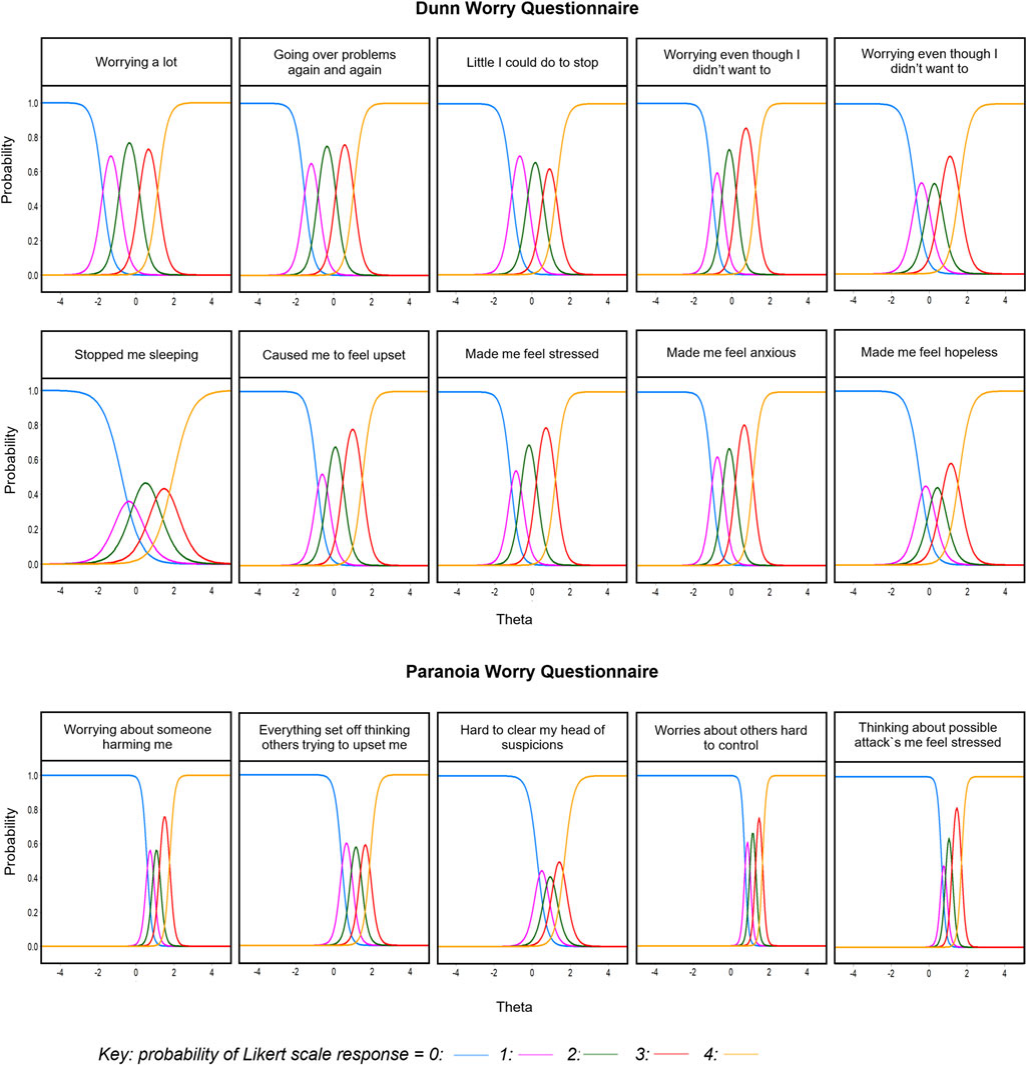
Category response curves (CRCs) for the Dunn Worry Questionnaire and Paranoia Worries Questionnaire. The lines represent the probability (*y* axis) of responding to each Likert scale option (0–4) across the distribution of *θ* (*x* axis) for each item.

All 10 general worry items and all five paranoia worry items selected from the derivation analysis had adequate item fit and residual correlations below 0.2 in the cross-validation sample. The IRT item parameters for both scales from this sample are shown in Table 1. Figure 1 shows the category response curves (CRCs) for each scale, depicting for each item the probability of every response option (0–4) along the distribution of *θ*. The discrimination parameter (*a*) is represented by the steepness of the curve, with higher values indicating a greater capacity to discriminate small differences in severity levels.

**Table 1.** IRT parameters for the final versions of the DWQ and PWQ with the cross-validation sample (*n* = 449)

Dunn Worry Questionnaire	*a*	*b*_1_	*b*_2_	*b*_3_	*b*_4_
1. I've been worrying a lot	3.81 (0.29)	−1.78 (0.12)	−0.89 (0.08)	0.1 (0.06)	1.15 (0.08)
2. In my mind I have been going over problems again and again	4.18 (0.32)	−1.56 (0.10)	−0.82 (0.07)	0.11 (0.06)	1.06 (0.08)
3. There was little I could do to stop worrying	4.02 (0.30)	−1.07 (0.08)	−0.22 (0.06)	0.57 (0.07)	1.29 (0.09)
4. I have been worrying even though I didn't want to	5.03 (0.40)	−1.06 (0.08)	−0.51 (0.06)	0.22 (0.06)	1.24 (0.08)
5. Worry has stopped me focusing on important things in my day	3.49 (0.26)	−0.76 (0.08)	−0.08 (0.06)	0.60 (0.07)	1.57 (0.11)
6. Worry has stopped me sleeping	1.98 (0.16)	−0.77 (0.09)	0.01 (0.08)	1.01 (0.10)	1.95 (0.16)
7. Worry has caused me to feel upset	4.11 (0.31)	−0.90 (0.08)	−0.34 (0.06)	0.47 (0.07)	1.49 (0.10)
8. Worry has made me feel stressed	4.24 (0.32)	−1.16 (0.09)	−0.59 (0.07)	0.22 (0.06)	1.23 (0.09)
9. Worry has made me feel anxious	4.92 (0.39)	−1.05 (0.08)	−0.46 (0.06)	0.21 (0.06)	1.12 (0.08)
10. Worry has made me feel hopeless	3.17 (0.24)	−0.50 (0.07)	0.11 (0.07)	0.71 (0.07)	1.55 (0.11)
Paranoia Worries Questionnaire	*a*	*b*_*1*_	*b*_*2*_	*b*_*3*_	*b*_*4*_
1. I've been worrying about someone trying to harm me	7.57 (0.94)	0.57 (0.06)	0.91 (0.06)	1.24 (0.07)	1.77 (0.10)
2. Anything and everything has set my mind thinking about people trying to upset me	5.45 (0.59)	0.41 (0.06)	0.91 (0.07)	1.40 (0.08)	1.89 (0.12)
3. It has been hard to clear my head of suspicions	4.10 (0.39)	0.27 (0.06)	0.74 (0.07)	1.15 (0.08)	1.69 (0.10)
4. Worries about someone trying to harm me have been really hard to control	10.7 (1.72)	0.73 (0.06)	1.00 (0.06)	1.29 (0.07)	1.66 (0.09)
5. Thinking about the possible attacks on me has made me feel stressed	9.17 (1.22)	0.66 (0.06)	0.89 (0.06)	1.22 (0.07)	1.72 (0.10)

Standard errors are shown in parentheses.

*a* = discrimination, *b* = difficulty parameters at the category thresholds between 0–1 (*b*_1_), 1–2 (*b*_2_), 2–3 (*b*_3_) and 3–4 (*b*_4_).

### Dunn Worry Questionnaire

All 10 items of the Dunn Worry Questionnaire (DWQ) had high levels of discrimination (*a* = 1.98–5.03), indicating an increase in latent worry leads to a high probability that each item will be endorsed. The most discriminating item was ‘*I have been worrying even though I didn't want to*’ (*a* = 5.03), suggesting endorsement of this item was particularly representative of more severe worry. The least discriminating item was ‘*Worry has stopped me sleeping*’ (*a* = 1.98), although this was still well above the threshold of 1.0 for a highly discriminative item (Baker and Kim, 2017). The CRCs in Fig. 1 show that all items of the DWQ discriminate well across a wide range of the worry distribution. Examination of the expected score across this distribution shows most people are likely to endorse a number of items on this scale; however, more severe worry is associated with higher levels of item endorsement.

**Fig. 2.**
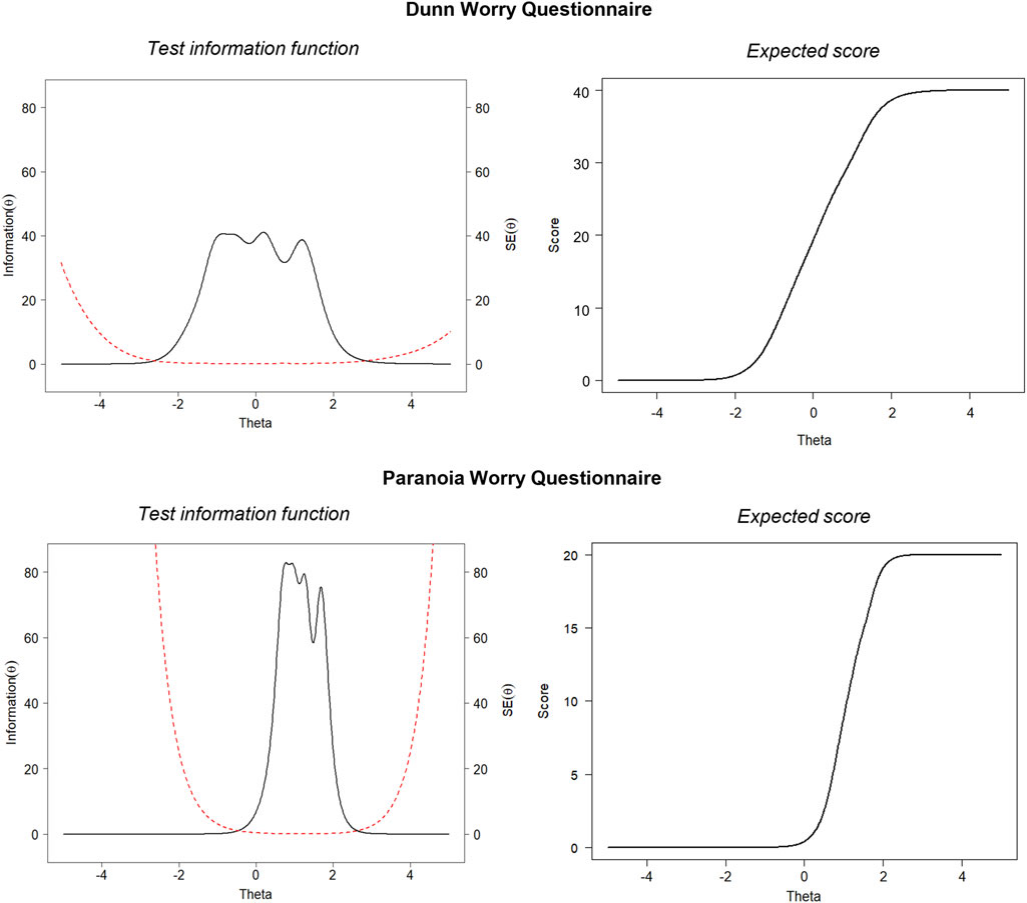
Test information (TI) with standard errors (----) and expected score across the *θ* distribution for DWQ and PWQ.

The TI function in Fig. 2 represents the reliability of the test at different points of the *θ* spectrum. The DWQ had excellent reliability across the worry distribution, providing equivalent *α* values above 0.95 (TI>20) between 1.5 s.d. below and 1.7 s.d. above the average levels of trait worry (Fig. 2). Precision was high within this range with extremely small standard errors (0.16–0.22). The reliability only dropped below *α* = 0.85 (TI = 6.67) after the extremes of 2.0 s.d. below and 2.1 s.d. above the average trait worry where standard errors increased. The maximum information obtained was 41.1 (s.e. = 0.16) at a *θ* level of 0.2, equivalent to a reliability of *α* = 0.98. The DWQ had excellent test-retest reliability over 1 week with an intra-class correlation of 0.97 (95% CI 0.95–0.98, *p* < 0.001) between the two time points. These findings demonstrate that the DWQ has an excellent ability to discriminate worry with high reliability and precision for use in both non-clinical and clinical populations.

### Paranoia Worries Questionnaire

The item parameters for the Paranoia Worries Questionnaire (PWQ) show the five selected items are extremely discriminative (*a* = 4.10–10.7). The item with the strongest discriminative power was ‘*Worries about someone trying to harm me have been really hard to control*’ (*a* = 10.7), suggesting endorsement of this item was the most indicative of severe paranoia worry. As shown in Fig. 1, all five items tended to discriminate at higher levels of *θ*. Indeed, the steep expected score function in Fig. 2 shows people at the average level (*θ* = 0) have a low probability of scoring above zero, whereas item endorsement is strongly indicative of severe paranoia worry.

The TI function in Fig. 2 confirms that overall the PWQ provides an extremely high level of information, but primarily across the higher end of the paranoia worry distribution. The PWQ had excellent reliability with equivalent *α* values higher than 0.95 within 0.29–2.05 s.d.s above the average levels of paranoia worry. Precision was also high in this range with extremely small standard errors (0.11–0.22). The maximum information obtained was 82.8 (s.e. = 0.11) at a *θ* level of 0.78, equivalent to a reliability of *α* = 0.99. Conversely, the PWQ items discriminate less well at the lower end of the spectrum, with little information obtained at *θ* values below zero. The reliability of the PWQ starts to drop below *α* = 0.85 after −0.01 s.d.s below and 2.27 s.d.s above the average levels of paranoia worry where standard errors rapidly increase (Fig. 2). Test-retest reliability over 1 week was excellent with an intra-class correlation of 0.96 (95% CI 0.95–0.98, *p* < 0.001). These findings suggest the PWQ is highly discriminative of severe levels of paranoia worry for use in clinical populations.

### Concurrent validity

The concurrent validity of the scales was demonstrated by the correlations with other measures of worry, anxiety and paranoia (Table 2). For both the non-clinical and persecutory delusion samples, the DWQ demonstrated a distinct pattern of strong correlations with related measures of worry (i.e. PSWQ, perseverative thinking, generalised anxiety and anxiety symptoms) and a moderate association with paranoia. Conversely, the PWQ was strongly related to paranoia and moderately correlated with measures of worry and anxiety.

**Table 2.** Bivariate correlations between the DWQ and PWQ with other measures in the cross-validation samples from the general population (*n* = 273) and patients with persecutory delusions (*n* = 79)

	General worries (DWQ)		Paranoia worries (PWQ)	
	*r*	*p*	*r*	*p*
General population group				
Paranoia worries (PWQ)	0.46	<0.001		
Worry (PSWQ)	0.73	<0.001	0.41	<0.001
Perseverative thinking (PTQ)	0.80	<0.001	0.48	<0.001
Generalised anxiety (GAD7)	0.72	<0.001	0.49	<0.001
Anxiety (DASS)	0.69	<0.001	0.52	<0.001
Paranoia (GPTS)	0.42	<0.001	0.80	<0.001
Persecutory delusion group				
Paranoia worries (PWQ)	0.69	<0.001		
Worry (PSWQ)	0.70	<0.001	0.47	<0.001
Perseverative thinking (PTQ)	0.63	<0.001	0.53	<0.001
Anxiety (BAI)	0.39	<0.001	0.42	<0.001
Paranoia (GPTS)	0.55	<0.001	0.73	<0.001

Further supporting the construct validity of each scale, there was a significant group effect on the DWQ [*F*_(3,441)_ = 97, *p* < 0.001] and PWQ [*F*_(3,441)_ = 255.5, *p* < 0.001] scores for the four groups (non-clinical, non-clinical GAD, psychosis, SAD) (see Table 3). As expected, the highest scores on the DWQ were observed in the GAD and persecutory delusion groups (mean difference = 1.4, *p* = 0.78). Participants in these two groups scored significantly higher (*p* < 0.001) than the remaining participants from the general population and patients with SAD. As would be expected, patients with SAD also scored significantly higher than those in the general population (*p* < 0.001). In line with the PWQ as a clinical measure of paranoid worry, patients with persecutory delusions scored significantly higher than all other subgroups (*p* < 0.001). Participants in the high GAD subgroup scored significantly higher (*p* < 0.001) than patients with SAD and general population controls, both of which had similarly low scores (*p* = 0.74).

**Table 3.** Mean total scores on the DWQ, PWQ, PSWQ and GPTS across all participant subgroups

	General worry (DWQ)	Paranoia worries (PWQ)	Worry (PSWQ)	Paranoid thoughts (GPTS)
General population				
Controls (*n* = 223)	12.9 (8.27)	0.91 (2.17)	41.0 (11.2)	19.5 (7.31)
GAD (*n* = 51)	28.7 (7.59)	4.18 (4.92)	62.9 (11.3)	30.1 (19.2)
Anxiety disorders (*n* = 97)	22.7 (9.00)	1.31 (2.66)	–	–
Persecutory delusions (*n* = 78)	27.3 (7.48)	12.7 (5.05)	61.6 (11.9)	56.7 (16.5)
Clinical follow-up (*n* = 43)				
Time 1	27.5 (8.03)	12.8 (4.95)	60.3 (13.1)	55.5 (16.6)
Time 2	25.0 (9.02)	11.4 (5.14)	59.4 (11.5)	51.4 (16.3)
Intervention subgroup (*n* = 12)				
Baseline	29.0 (5.01)	14.8 (3.35)	62.4 (9.26)	62.4 (9.52)
8-week follow-up	22.8 (9.45)	9.92 (3.70)	54.0 (8.63)	47.0 (16.5)

Standard deviations in parentheses.

### Sensitivity to change

Forty-three patients with persecutory delusions (mean age = 41.0, s.d. = 10.6, female = 28%, male = 72%) repeated the questionnaires either 8 (*n* = 27), 16 (*n* = 14) or 24 weeks (*n* = 2) later. Across this whole group (which comprised both randomisation arms of the trial), changes in the DWQ were moderately correlated with changes in the PSWQ (*r* = 0.50, *p* < 0.001) and the general psychopathology subscale of the PANSS (*r* = 0.34, *p* = 0.028), although overall there was no significant change in worry on either the DWQ (ES = 0.31, *p* > 0.05) or the PSWQ (ES = 0.07, *p* > 0.05). Twelve participants completed the assessments directly before and after the worry treatment. In these participants, reductions in worry were observed for the DWQ (ES = 1.2, *v* = 54, *p* = 0.03) and the PSWQ (ES = 0.9, *v* = 74.5, *p* = 0.003). Using the RCI, 8/12 participants showed a reliable reduction in DWQ worry scores following the intervention (online Supplementary materials). Notably, four of these patients did not show corresponding reliable changes on the PSWQ (only five participants showed a significant reduction on this scale). Only one participant with a significant RCI on the PSWQ did not show a significant change on the DWQ. These findings indicate the DWQ might have greater sensitivity to reductions in worry compared with the PSWQ.

Changes on the PWQ were strongly correlated with changes in GPTS paranoia (*r* = 0.61, *p* < 0.001) and DWQ worry scores (*r* = 0.74, *p* < 0.001), although there were no significant changes between the two time points in PWQ paranoid worry (ES = 0.29, *p* > 0.05) or GPTS paranoid thoughts (ES = 0.25, *p* > 0.05) scores. Notably, the correlation between change in paranoid worry and worry measured by the PSWQ (*r* = 0.42, *p* = 0.005) was not as strong as the correlation with the DWQ. In the subgroup of 12 participants who received the worry intervention between the measurements, large changes were observed in both paranoid worry (ES = 1.45, *v* = 66, *p* = 0.002) and paranoid thoughts (ES = 1.6, *v* = 63, *p* = 0.004). Following the intervention, the PWQ was able to detect reliable reductions in paranoid worry in 9/12 patients, which corresponded with reliable improvements in paranoia as measured by the GPTS.

### Clinical cut-off scores

The ROC curves for both questionnaires and the sensitivity and specificity at different thresholds are shown in the online Supplementary materials.

#### Dunn Worry Questionnaire

The ROC analysis for the DWQ provided an AUC of 0.90 (95% CI 0.86–0.95), demonstrating an excellent level of discriminatory power. This indicates that a person with clinically identified levels of worry is 90% more likely to have a higher score on the DWQ than someone in the general population. This analysis identified the closest threshold to the optimal cut-off point was a score of 21 or above, providing a sensitivity of 0.88 (95% CI 0.80–0.95) and specificity of 0.83 (95% CI 0.78–0.87).

#### Paranoia Worries Questionnaire

The PWQ had an excellent ability to discriminate non-clinical and clinical levels of paranoia worry with an AUC of 0.95 (95% CI 0.90–0.98). This indicates a person with a clinically diagnosed persecutory delusion is 95% more likely to have a higher PWQ score than someone in the general population. The ROC analysis identified the closest threshold to the optimal cut-off point was a score of 5 or above, providing a sensitivity of 0.91 (95% CI 0.84–0.96) and specificity of 0.89 (95% CI 0.85–0.92). This same threshold was identified when the analysis was repeated with the SAD group as controls (AUC = 0.96, 95% CI 0.93–0.99), where a score of 5 provided a sensitivity of 0.91 (95% CI 0.84–0.97) and a specificity of 0.91 (95% CI 0.84–0.96). This demonstrates that even within clinical populations, a PWQ score of 5 or above is highly indicative of severe paranoia worry in the context of persecutory delusions.

## Discussion

It is increasingly recognised that mental health conditions arise from multiple interacting factors that cross diagnostic boundaries. Worry is a plausible contributory factor to many mental health conditions, as shown in a recent analysis of epidemiological survey data using a dynamic Bayesian network approach (Kuipers *et al*., 2019). Our clinical and research experience is that the assessment of worry can be improved. Therefore, we developed a new scale of general problematic worry, combining classical test theory with latent trait models, that has a clear time period, is straightforward to complete and includes the impact of the thinking style. The IRT analysis shows that the DWQ reliably assesses the range of worry severity across the non-clinical and clinical population and can discriminate between different levels of this spectrum. Internal reliability and test-retest reliability were extremely high. Sensitivity to change was established and convergent validity was shown with existing assessments of worry, perseverative negative thinking and GADs. As would be expected, individuals from the general population meeting cut-offs for GAD and patients with persecutory delusions scored more highly on the DWQ than patients with SAD, who had higher scores than non-clinical controls. A psychometrically strong, comprehensible measure of problematic worry has been produced.

In a clear illustration of the trans-diagnostic importance of worry, we have shown in the WIT that treating worry in patients with psychosis leads to a reduction in persecutory delusions (Freeman *et al*., 2015). The best treatment approaches regularly monitor the key outcome. We therefore also developed a brief measure of problematic worry focused on paranoid content, the PWQ. In contrast to the DWQ, reliable across non-clinical and clinical levels of worry, the PWQ is most reliable for those at the clinical end of the spectrum. The items are primarily discriminative of severe levels of paranoia worry, which makes it ideal for the intended use in treatment with patients with psychosis. To score on the measure requires both paranoid fears and worry. The scale has extremely high internal reliability at severe levels of paranoia worry and excellent test-retest reliability. It is associated with scores on assessments for paranoia in particular but also negative repetitive thinking. In the context of the treatment trial, the PWQ showed sensitivity to clinical change.

There are limitations in the development of the questionnaires. The questionnaires were not tested with patients with GAD, which is considered the archetypal disorder of worry. It would be valuable for the questionnaires to be tested with this patient group. However, individuals with persecutory delusions, who were tested in this study, typically have levels of worry comparable to patients with GAD (Freeman and Garety, 1999). Both the non-clinical individuals screening positive for GAD and the patients with persecutory delusions scored highly on the new general worry measures. We think it highly unlikely that a different pattern would be found for patients with GAD. The development of the assessments would have benefited from greater input from patients. Patients only gave feedback on the ease of completion of the initial item pool and subsequent scales. In the years since the development of the measures we have developed much more rigorous patient involvement procedures. Further, the participant groups, clinical and non-clinical, were unlikely to have been fully representative of the populations from which they were drawn. The true potential of the questionnaires will only be known with use.

## Supplementary material

For supplementary material accompanying this paper visit http://dx.doi.org/10.1017/S0033291719000588. click here to view supplementary material

## Appendix

**1. The Dunn Worry Questionnaire**

Please circle the numbers that best describe your experience *in the past month*.

**Table:**

	None of the time	Rarely	Some of the time	Often	All of the time
I've been worrying a lot	0	1	2	3	4
In my mind I have been going over problems again and again	0	1	2	3	4
There was little I could do to stop worrying	0	1	2	3	4
I have been worrying even though I didn't want to	0	1	2	3	4
Worry has stopped me focussing on important things in my day	0	1	2	3	4
Worry has stopped me sleeping	0	1	2	3	4
Worry has caused me to feel upset	0	1	2	3	4
Worry has made me feel stressed	0	1	2	3	4
Worry has made me feel anxious	0	1	2	3	4
Worry has made me feel hopeless	0	1	2	3	4

**2. The Paranoia Worries Questionnaire**

The following items concern worries you may have about others trying to upset or harm you.

Please circle the numbers that best describe your experience *in the past month*.

**Table:**

	None of the time	Rarely	Some of the time	Often	All of the time
I've been worrying about someone trying to harm me	0	1	2	3	4
Anything and everything has set my mind thinking about people trying to upset me	0	1	2	3	4
It has been hard to clear my head of suspicions	0	1	2	3	4
Worries about someone trying to harm me have been really hard to control	0	1	2	3	4
Thinking about the possible attacks on me has made me feel stressed	0	1	2	3	4
