# Supplementary material for: The Dunn Worry Questionnaire and the Paranoia Worries Questionnaire: new assessments of worry
Source: Psychol Med. 2019 Apr 5;50(5):771–80. doi: 10.1017/S0033291719000588 (PMC7168652; doi:10.1017/S0033291719000588)
Supplement: Supplementary file 1 [file S0033291719000588sup001.docx]

**SUPPLEMENTARY MATERIALS**

Table 1. IRT parameters and factor loadings for general worry items in derivation sample.

| **General worry items** | **Factor loading** | ***a*** | ***b*1** | ***b*2** | ***b*3** | ***b*4** |
| --- | --- | --- | --- | --- | --- | --- |
| I’ve been worrying a lot * | 0.92 | 3.09 | -1.27 | -0.33 | 0.67 | 1.59 |
| In my mind I have been going over problems again and again * | 0.85 | 2.97 | -1.06 | 0.12 | 0.69 | 1.64 |
| I kept thinking about all the things that could go wrong in my life | 0.82 | 2.91 | -0.61 | 0.23 | 1.04 | 1.77 |
| My days have felt taken over by worry | 0.74 | 4.28 | -0.07 | 0.55 | 1.33 | 1.93 |
| I’ve worried about everything | 0.79 | 3.24 | 0.08 | 0.89 | 1.55 | 2.12 |
| I have worried too much | 0.91 | 3.00 | -0.77 | -0.02 | 0.80 | 1.76 |
| Mistakes, dangers, and disasters have been on my mind | 0.60 | 2.20 | -0.48 | 0.43 | 1.18 | 2.07 |
| My worrying has felt out of control | 0.77 | 4.88 | 0.09 | 0.56 | 1.29 | 1.80 |
| There was little I could do to stop worrying * | 0.79 | 3.62 | -0.24 | 0.54 | 1.17 | 1.98 |
| Worry has caused me to feel upset * | 0.83 | 3.36 | -0.60 | 0.10 | 0.93 | 1.78 |
| Worry has made me feel stressed * | 0.94 | 3.56 | -0.77 | -0.08 | 0.78 | 1.59 |
| I have felt sick with worrying | 0.60 | 2.70 | 0.30 | 0.95 | 1.85 | 2.23 |
| Worry has made me feel anxious * | 0.85 | 3.33 | -0.52 | 0.18 | 0.96 | 1.97 |
| Worry has made me feel hopeless * | 0.73 | 3.23 | -0.15 | 0.46 | 1.10 | 1.92 |
| Worry has stopped me focussing on important things in my day * | 0.78 | 2.95 | -0.35 | 0.33 | 1.15 | 1.97 |
| Worry has stopped me sleeping * | 0.69 | 1.62 | -0.44 | 0.57 | 1.66 | 3.03 |
| I have been worrying even though I didn’t want to * | 0.89 | 3.24 | -0.68 | 0.02 | 0.91 | 1.68 |
| Fears have been playing on my mind | 0.80 | 3.14 | -0.49 | 0.22 | 1.01 | 2.02 |
| I felt driven to keep thinking about the things that worry me | 0.77 | 3.27 | -0.25 | 0.43 | 1.16 | 2.09 |
| My mind could not rest because of all my worries | 0.79 | 4.28 | -0.35 | 0.29 | 1.08 | 1.79 |
| Worry has got in the way of my relationships with friends and family | 0.65 | 2.52 | 0.00 | 0.54 | 1.53 | 2.28 |
| **Items excluded from IRT analysis** |  |  |  |  |  |  |
| I’ve been a worrier | 0.97 |  |  |  |  |  |
| Worrisome thoughts kept going round in my head | 0.87 |  |  |  |  |  |
| I have spent a lot of time worrying about the worst happening to me | 0.69 |  |  |  |  |  |
| I’ve been thinking about how things will go wrong | 0.78 |  |  |  |  |  |
| Even small things made me worry | 0.73 |  |  |  |  |  |
| It didn’t take much to get me worried | 0.79 |  |  |  |  |  |
| Worrying has been making my problems seem bigger than they are | 0.90 |  |  |  |  |  |
| I’ve been thinking about problems but without arriving at any solutions | 0.77 |  |  |  |  |  |
| Once I started worrying it has been hard to stop | 0.85 |  |  |  |  |  |
| My worries have taken control of me | 0.67 |  |  |  |  |  |
| Worry has been causing me to be anxious | 0.89 |  |  |  |  |  |
| I’ve been anxious with worry | 0.85 |  |  |  |  |  |
| I have felt fed up of worrying | 0.88 |  |  |  |  |  |
| Worrying has made me tense | 0.90 |  |  |  |  |  |
| Worry has made me feel sad | 0.84 |  |  |  |  |  |
| It has been hard to concentrate because of worrying | 0.85 |  |  |  |  |  |
| I could not manage my tasks because my mind kept coming back to my worries | 0.77 |  |  |  |  |  |
| Worry has stopped me working efficiently | 0.72 |  |  |  |  |  |
| Upsetting images have been playing on my mind ^1^ | / |  |  |  |  |  |

Note: a = discrimination, b = difficulty parameters at the category thresholds between 0-1 (b1), 1-2 (b2), 2-3 (b3) and 3-4 (b4).
^1^ item deleted from EFA due to cross-loading over 0.3 with paranoid worry factor

Table 2. IRT parameters and factor loadings for paranoia worry items in derivation sample.

| **Paranoia worry items** | **Factor loading** | ***a*** | ***b*_1_** | ***b*_2_** | ***b*_3_** | ***b*_4_** |
| --- | --- | --- | --- | --- | --- | --- |
| I’ve been worrying about someone trying to harm me * | 0.90 | 6.39 | 0.71 | 1.03 | 1.46 | 1.84 |
| Anything and everything has set my mind thinking about people trying to upset me * | 0.85 | 6.24 | 0.70 | 1.04 | 1.44 | 1.80 |
| I’ve not been able to stop thinking about people getting at me. | 0.97 | 10.0 | 0.68 | 1.02 | 1.34 | 1.67 |
| It has been hard to clear my head of suspicions * | 0.71 | 4.33 | 0.56 | 0.94 | 1.27 | 1.75 |
| I have been thinking a lot about someone being hostile towards me. | 0.80 | 4.70 | 0.41 | 0.89 | 1.37 | 1.88 |
| I have been thinking a lot about the attacks on me. | 0.97 | 8.38 | 0.78 | 1.10 | 1.47 | 1.78 |
| Worries about someone trying to harm me have been really hard to control * | 1.00 | 9.57 | 0.80 | 1.13 | 1.36 | 1.81 |
| Thinking about the possible attacks on me has made me feel stressed. * | 0.94 | 7.05 | 0.76 | 0.99 | 1.41 | 1.74 |
| Fears about harm from another person have been going round and round my mind. | 0.97 | 8.29 | 0.73 | 1.04 | 1.44 | 1.68 |
| **Items excluded from IRT analysis** |  |  |  |  |  |  |
| My mind has been full of thoughts of someone getting at me | 0.82 |  |  |  |  |  |
| Thoughts about someone threatening me have been going round my mind | 0.89 |  |  |  |  |  |
| Suspicions have been going around my head | 0.68 |  |  |  |  |  |
| Worries about what someone is doing to me have been really upsetting me | 0.82 |  |  |  |  |  |
| I have been thinking a lot about bad rumours about me. | 0.62 |  |  |  |  |  |
| It’s been hard to sleep because of thinking about my fears ^1^ | / |  |  |  |  |  |
| Whether others will be nasty to me has been in my thoughts a lot ^1^ | / |  |  |  |  |  |

Note: a = discrimination, b = difficulty parameters at the category thresholds between 0-1 (b1), 1-2 (b2), 2-3 (b3) and 3-4 (b4).
^1^ item deleted from EFA due to cross-loading over 0.3 with paranoid worry factor

|  | **DWQ** | | | **PSWQ** | | | **PWQ** | | | **GPTS** | | |
| --- | --- | --- | --- | --- | --- | --- | --- | --- | --- | --- | --- | --- |
| ID | Pre | Post | RCI | Pre | Post | RCI | Pre | Post | RCI | Pre | Post | RCI |
| 1 | 29 | 7 | -10.4* | 55 | 39 | -4.07* | 15 | 6 | -6.33* | 65 | 26 | -13.0* |
| 2 | 28 | 22 | -2.82* | 67 | 62 | -1.27 | 12 | 6 | -4.22* | 51 | 38 | -4.32* |
| 3 | 28 | 28 | 0.00 | 51 | 56 | 1.27 | 15 | 15 | 0.00 | 61 | 66 | 1.66 |
| 4 | 31 | 35 | 1.88 | 71 | 70 | -0.25 | 19 | 18 | -0.70 | 72 | 72 | 0 |
| 5 | 39 | 21 | -8.47* | 80 | 54 | -6.62* | 20 | 11 | -6.33* | 77 | 64 | -4.32* |
| 6 | 27 | 9 | -8.47* | 54 | 50 | -1.02 | 13 | 6 | -4.93* | 67 | 55 | -3.99* |
| 7 | 29 | 17 | -5.65* | 69 | 56 | -3.31* | 13 | 10 | -2.11* | 43 | 45 | 0.66 |
| 8 | 24 | 21 | -1.41 | 52 | 42 | -2.55* | 15 | 9 | -4.22* | 60 | 35 | -8.30* |
| 9 | 35 | 33 | -0.94 | 59 | 53 | -1.52 | 20 | 10 | -7.04* | 64 | 45 | -6.31* |
| 10 | 19 | 37 | 8.47* | 55 | 49 | -1.53 | 11 | 8 | -2.11* | 64 | 25 | -13.0* |
| 11 | 31 | 25 | -2.82* | 69 | 63 | -1.53 | 15 | 12 | -2.11* | 71 | 62 | -2.99* |
| 12 | 28 | 19 | -4.24* | 67 | 54 | -3.31* | 10 | 8 | -1.41 | 54 | 31 | -7.64* |

**Reliable change index (RCI)**
Table 3. RCIs of the difference between baseline and 8-week follow up for participants who received the worry intervention.

* RCI greater than +/- 1.96 indicates a significant change in score (*p*<.05) between the two time points given the reliability of each measure.

**ROC analysis**


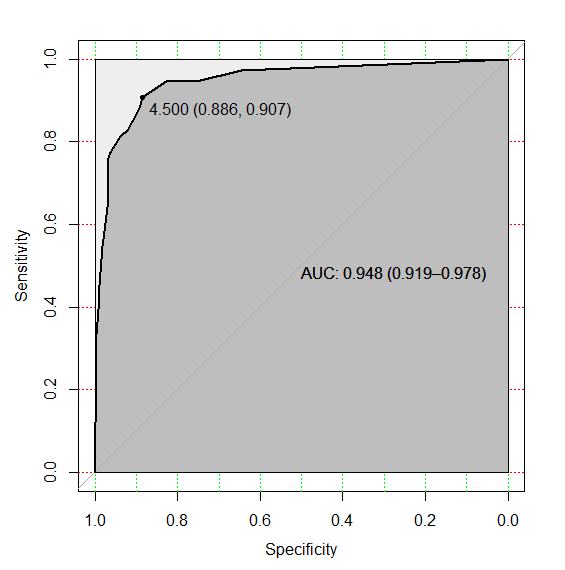

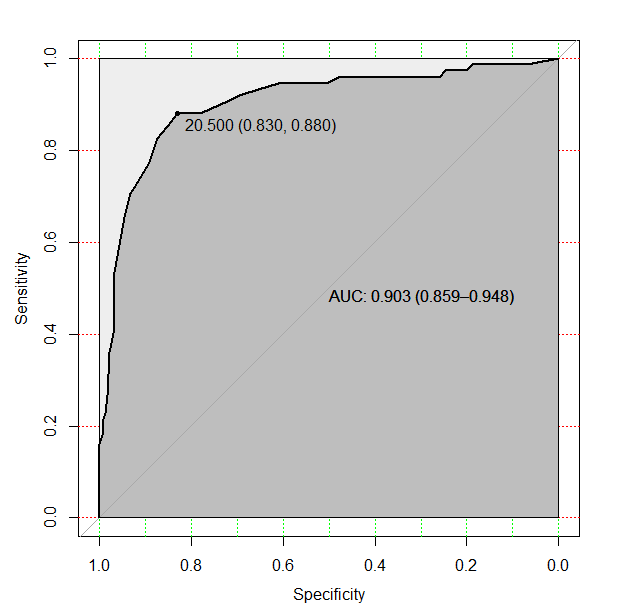
Figure 1. ROC curves for a) Dunn Worry Questionnaire and b) Paranoia Worries Questionnaire. The area under the curve (AUC) is shown with 95% CIs. The optimal cut-off threshold is marked at the upper left of each curve with specificity and sensitivity in parentheses.

sTable 4. Sensitivity and specificity for different total score cut-offs for the GWS and SWS. Optimal cut-offs in bold and 95% confidence intervals in parentheses.

| **Total Score** | **Sensitivity (Se)** | **Se 95% CI** | **Specificity** | **95% CI** |  |
| --- | --- | --- | --- | --- | --- |
| *Dunn Worry Questionnaire* | |  |  |  |  |
| 19 | 0.91 | 0.84-0.96 | 0.72 | 0.65-0.78 |  |
| 20 | 0.88 | 0.80-0.95 | 0.78 | 0.73-0.83 |  |
| **21** | **0.88** | **0.81-0.95** | **0.83** | **0.78-0.87** |  |
| 22 | 0.85 | 0.77-0.93 | 0.85 | 0.80-0.90 |  |
| 23 | 0.83 | 0.73-0.91 | 0.87 | 0.83-0.92 |  |
| *Paranoia Worries Scale* | |  |  |  |  |
| 4 | 0.95 | 0.90-0.99 | 0.84 | 0.79-0.88 |  |
| 5 | 0.94 | 0.87-0.99 | 0.86 | 0.81-0.90 |  |
| **6** | **0.92** | 0.86-0.97 | **0.88** | **0.84-0.92** |  |
| 7 | 0.90 | 0.82-0.96 | 0.89 | 0.85-0.92 |  |
| 8 | 0.90 | 0.83-0.96 | 0.89 | 0.86-0.93 |  |
